# Report of Discussions of the Missisippi Valley Association

**Published:** 1856-04

**Authors:** 


### REPORT OF DISCUSSIONS OF THE MISS. VALLEY ASSOCIATION.

THURSDAY, Feb. 21st, 1856.

Second day, morning session. The Association resumed the discussion of the questions proposed for consideration by the executive committee. The next in order was the

3D. QUESTION. What is the best means of drying cavities previous to filling?

Dr. Taylor said it had been his habit to dry out cavities with dry cotton. Within the last year he had used a tissue paper made specially for this purpose, called the French bibulous paper. He had previously used the paper coming with the foil. The French paper is much better.

Dr. Bonsal had tried this paper and liked it very much. Cotton left a glazed surface, which the paper did not.

Dr. Smith had been in the habit of using the paper coming round the foil, often cotton, and succeeded in drying his cavities very well.

Dr. Ulrey thought the French paper dried the cavity much better than cotton, and should always use it if he could obtain it.

Dr. Watt had not used the tissue paper much, had tried buck-skin shavings, but had succeeded best with the warm air bath, or the projection of a stream of warm air into the cavity of the tooth. The most convenient method of doing this was to use a small blow-pipe in which is a cylinder of brass or other material capable of retaining heat, and which can be heated over a spirit-lamp. The air can be projected through by attaching a smooth indian rubber bag to the outer end of the pipe, with a hole in it to admit the air, and which can be covered by the thumb when the pressure is given. Care must be taken not to get the stream of air too hot. The effect of this stream of warm air on the appearance of the cavity was remarkable. It resembled a dried bone. The canister once heated would retain sufficient heat for eight or ten minutes. With this apparatus, he did not care much about the particular method of wiping out.

Dr. Taft had used this warm air apparatus and liked it much. It was very cenvenient in drying cavities which become wet when partly filled. It was impossible in such cases to wipe them dry on account of the irregularities of the surface, and nothing but a stream of hot air would answer. It also answered a valuable purpose in drying gold covered with moisture before the cavity is full. In these respects it answers a purpose not otherwise obtained. It is also valuable in drying out cavities before filling. Some breaths are so moist that it is impossible to make the gold consolidate. The patient should be instructed to breathe through the nostrils. A good plan to keep the saliva from flowing into the cavity was to place a napkin on the tongue and to close the ducts by placing blotting paper over them. A great help in preventing the flow of saliva was to keep the jaw, tongue, and cheeks all still. There was a great difference among patients in this respect, also in the quantity and quality of the saliva. In some persons it resembled an oily mucus, and was drawn up by capillary attraction.

Dr. Griffith had used various means to prevent the saliva from flowing into the cavity. Much depended on keeping the tongue still and thus prevent the accumulation of saliva.

He always got the foil all ready, wiped the cavity out quickly and filled readily.

Dr. Goddard said he depended much on the use of napkins and watch-springs to prevent the accumulation of saliva. He had a gross or more of linen napkins of all sizes, from two inches by four, up to a common sized handkerchief. After preparing his cavity, he took a piece of watch-spring four or six inches long, and wrapped a small napking around it, and placed it under the tongue around against the teeth. Another similarly wrapped was placed between the teeth and the cheeks. He sometimes wrapped a small coil, and placed one side against the teeth and the other against the cheek to keep them apart. He also placed a napkin on the tongue, and had the patient place a finger on it. These means prevented the accumulation of saliva by closing the ducts and absorbing what was in the mouth. He had his foil all prepared and rolled in slips, wiped out with cotton, and filled quickly. If any saliva got in he emptied the cavity and began again. He used a golden shield placed in the corner of the mouth for his instruments to work through.

Dr. Horton said he generally used cotton to dry the cavity, sometimes used paper. He used napkins in the mouth. He found it beneficial to rinse the mouth with water, it prevents the flow of saliva. If the filling got wet, he always began anew.

Dr. Taylor said he had found that linen napkins absorbed more readily than cotton. He used a speculum to cover the tongue. Much depended on the rapidity with which the gold is introduced. He thought it impossible to commence with the gold in strips and fill large cavities fast enough to get done before the accumulation of saliva would overflow the cavity. His plan was to have all things ready, dry quickly, have gold in bunches large enough to fill the cavity, and fill immediately.

Dr. Bonsall said he had found birds-eye linen better than other kind for absorption.

Dr. Goddard said that partly worn was better than new.

Dr. Griffith said there were some portions of a cavity not yet mentioned, a cavity when the mouth was very small, the cheek full and hard, also when the cavity was on a level with the gum in the lower jaw. Such cavities he had never been able to perfectly dry.

Dr. Taylor thought a napkin pressed hard against the adjoining parts would answer the purpose.

Dr. Goddard said his plan of springs and napkins would keep them dry.

Dr. Horton thought absolute dryness in such cases not so essential. He had operated in such a case, and the filling had remained in three and a-half years.

Dr. Griffith said he had had them remain ten years.

4TH. QUESTION. Has Block work any advantages over single teeth, and if so what are they?

Dr. Griffith said he had no experience in block work.

Dr. Goddard used generally single teeth.

The rest of the members generally used single teeth.

5TH QUESTION. What are the best methods of, and the best instruments for separating teeth preparatory to filling?

Dr. Griffith said he used a small file, very thin and very fine. He so used it as to make the space cut much larger on the inside, and filled from the inside. He never wedged.

Dr. Bonsall's plan was similar to Dr. Griffith's. He occasionally used a wedge to assist a fine saw.

Dr. Goddard said' for separating teeth, he generally used a chisel, except in doubtful cases, when he used a wedge to ascertain the extent of the defect. He used a file only after the tooth was filled, to smooth all asperities.

Dr. Bray practiced both wedging and filing according to circumstances. In young persons with open teeth, he generally wedged, in other cases filed.

Dr. Horton gave the preference to the file, though he used the chisel. He used a file partly worn out.

Dr. Hamlin used the file and wedge equally.

Dr. Richardson said that in operating on very sensitive teeth, he found less pain resulted from cutting than filing.

Dr. Ulrey thought we must be governed by the circumstances of the case. If the file accomplished the desired object he gave it the preference. In sensitive teeth, cutting was better. In young persons, he prefered wedging to cutting.

Dr. Goddard knew the file was generally used, but the question was, is it the best instrument. There were cases in which cutting was better. He had opened teeth in persons forty-five and fifty years of age by rubber wedges.

Dr. Ulrey wanted to know if any serious danger would result from wedging teeth properly.

Dr. Griffith said he had heard of suffering in several cases, and had seen one when much inflammation resulted, and the teeth became very sore. In this case, the teeth were all regular and close together. When the cavity was large, the edges of the tooth were generally so thin, that they had to be cut away till the edges became thick enough to hold the filling. It was a nice point how far to go in this matter, owing to different constitutions.

Dr. Bonsall thought with judicious management no injury could result from wedging, he had tried it considerably.

Dr. Horton wished to know if any members of the Association knew of cases in which teeth had to be extracted in consequence of inflammation resulting from wedging.

No one knew of any such case.

Dr. Keely had always found it best to produce the requisite space by wedging as quickly as possible,—say in one day. Young persons were apt to pull the wedges out when they felt uncomfortable.

Dr. Ulrey thought the quicker the process was done the more certain the inflammation.

The President said he used wedges in young persons when the edges were thick enough to hold the foil. Inflammation was caused by hurrying the process too fast. In the case mentioned by Dr. Griffith, the teeth were regular and close, and the pressure was felt on the whole arch, and it all had to move. Had the teeth been open, no such result would have happened.

Dr. Keely said he had found no bad result from wedging quickly, still he thought the file a very good institution.

Dr. Ramsey said he wedged with cotton gradually, where other means were reqnired he considered cutting better than the file.

Dr. Horton inquired of Dr. Griffith, how he cleaned his file while at work.

Dr. Griffith said he always dipped his file in water every few strokes. In cold weather he used warm water. If he thought it best to file, he filed and if his patients did not like it they could go somewhere else.

Dr. Taft said we must be governed by the circumstances of the case. When the patient was not too old, had a good constitution, and there was a space between the adjoining teeth, it was best to wedge. Sometimes it was necessary to cut and wedge both, as when the cavity is large and the edge thin. We cannot tell the size of the cavity and thickness of the edge in some case without wedging. When the teeth stood close they must be cut. When much cutting is required, a heavy cutting instrument is better than a file. It works quicker does not cause so much pain, and is not so liable to produce bad effects. After the requisite space was cut away the file was necessary to produce a smooth surface. He never used a file to produce a separation, as it was apt to cut the neighboring teeth.

Dr. Griffith wished to ask those favorable to cutting and wedging, whether, when a slight decay was perceptible, if it was not better to file it out, than wait till is was large enough to fill, and then cut or wedge. In such cases he filed, cutting more away on the inside than outside.

Dr Ulrey. said in such cases, more was cut away than is decayed. It was better to wedge and fill.

Dr. Taylor said it was hard to lay down positive rules. In many teeth, on account of the necks being narrower than the ends, it was impossible to file them away so that the filed surfaces would not come together again.

The Association then adjourned to 2 o'clock P. M.

SECOND DAY. THURSDAY.

AFTERNOON SESSION.—Association met at two o'clock, the President in the Chair.

The question, How can the color of natural teeth that require filling be most effectually preserved? was then taken up and discussed.

Dr. BONSALL stated that where the nerve had not been exposed, there was no danger of a change of color. He had sometimes used some substance of a light color, as a thin piece of horn or oiled silk, in order to prevent the gold from showing through where there was little except the enamel left, but the result had not been satisfactory. The only reliable method that he had found of filling such teeth was to clean the cavity out carefully, and fill even to the end of the fang with gold.

Dr. GRIFFITH had never tried to hide the gold. He thought it better to show the gold in a healthy tooth than by trying to hide it, to introduce some destructible substance and thus render the tooth unhealthy.

Dr. GODDARD had never tried to hide the gold. He doubted whether it could be done.

Dr. TAFT spoke of the various methods that had been used for this purpose, as the introduction of horn—of paper—and of plaster of Paris; but he had never experimented with any of them. Properly inserted fillings had sometimes become black after a certain length of time. He was at a loss to understand the cause.

Half-past two o'clock having been fixed for the election of officers, the discussion was suspended till after the balloting.

DISCUSSION RESUMED.

Question 7, was then taken up and discussed.

7th QUESTION. "Is gutta percha reliable as a base for artificial teeth."

Dr. JAMES TAYLOR said; gutta percha had been recommended only for temporary purposes, and for these he thought it reliable. He had been using it for the last few months, and in all cases but one it had answered the purpose. The only difficulty he had found in using it for temporary sets was in cases where the gum had not yet receded enough to allow a sufficient amount of it to be used. In certain cases he found it very useful. As for instance, he had a number of patients requiring an entire upper set, but who having a few good teeth in the under jaw were not willing to sacrifice them, and wanted something put in temporary until those teeth should decay, and a plate be required. For such temporary work gutta percha was just the thing.

In one case in which he had used it he had not succeeded in getting a good fit. To remedy this he heated the set in hot water, put it in the mouth and had the teeth closed upon it, when it adapted itself to the parts, fitted well and became very comfortable. It was so much more comfortable to wear than a metallic plate, that some of his patients preferred to go to the expense of having a new set every three or four years, to wearing gold.

There were however four objections to it for permanent work. 1st. It was bungling in the mouth. 2d. It was difficult to polish. 3d. It would after awhile give a little around the tooth. 4th. Tobacco would stain it. Still he did not doubt but gutta percha was destined to be entensively used by the profession. He regarded it as in its infancy, and it would no doubt be greatly improved. It supplied, like continuous gum work certain wants heretofore severely felt in the profession.

Dr. GODDARD had used gutta percha, and liked it exceedingly. It adhered better to the gum than a metallic plate.

He hoped it would be improved to become just what they all wanted.

Dr. Bonsall had put in but one set on gutta percha, but the experience of that one patient was highly favorable.

Dr. Ulrey said: It seemed to him the very thing for temporary work, but how long it would endure without changing its color he could not tell. He did not doubt but it would be very much improved, but was satisfied with it for temporary work just as it was, as compared with other materials. It made a more natural looking gum than the continuous gum work of Dr. Allen, as the polish of that made it a reflector and gave it an unnatural appearance, while this had a very natural look.

Dr. Taft thought its ability to accommodate itself to the parts of the mouth a great advantage. When from a change in the mouth, or other cause it failed to fit, it could be warmed over a spirit lamp, drawn up to the parts and made to fit perfectly. It was also found quite firm enough to hold the teeth in their place, but its adaptation to the parts was its great quality.

Dr. Taylor then described the manner of using it. He first fitted a sheet of gutta percha to the plaster model, but did not swedge it up. At first he had imagined that something would be gained in density and fit by swedging, but found by experience no such advantages, and had given up the practice. He then prepared a narrow plate, little or no wider than the teeth, and fitted it to the gutta percha, having it sufficiently hot to make it stick.

Ordinary plate teeth were not at all adapted to be used with gutta percha. As the whole of the metal is covered, silver answers just as well as gold. They were sometimes made with air chambers, but they adhere just as well without. One great advantage of their elasticity was, that in biting on one side the other was not so liable to fall, as was the case with the metallic plate. It yielded sufficiently to retain its position on the opposite side.

Question 8, was then taken up and discussed.

8th Question. "Can artificial teeth when moulded on a porcelain base ever be worn with advantage?"

Dr. Taylor said, as limited as his experience was he knew enough of the porcelain to satisfy him that the question might be answered in the affirmative. In a difficult case he after several trials with metallic plates, had ordered a set from Dr. Satterthwaite, and it adhered better than any plate he had put in.

Dr. Horton, of Cleveland, said that as he lived in the same city with Drs. Loomis and Wright, manufacturers of these porcelain teeth, he had had opportunities of knowing something about their work. That porcelain could be used as a base to great advantage in some cases there was no doubt, but he did doubt whether it would ever come into general use. In his neighborhood it had been puffed wonderfully, in fact, it had been a regular blowing and striking operation. One difficulty was that the cooling, after the set was taken from the furnace, was apt to cause a shrinkage and spoil the fit. This was remedied by grinding out. He had recommended a patient of his, whose case was a difficult one, to have a set, and he ordered it. This was not ground out, but fitted so well that no man could pull it off. He was able to masticate the toughest boarding house beef with it, and it remained perfectly clean. There was nothing about it that indicated any absorption. This patient had objected to a metallic plate on account of the taste in his mouth. The porcelain had remedied this, and proved fully satisfactory in other respects. Dr. Satterthwaite of Allen co., O., also manufactured these, and his did not seem liable to shrink.

An Essay on the various pathological conditions of inflamed Dentine was then read by Dr. Hamill, as an introduction to the discussion of the next question.

Evening Session.—Association met at 7 o'clock.

Subject No. 9, under Miscellaneous business, was then taken up and discussed.

Subject No. 9. What are the various pathological conditions of inflamed dentine, and what circumstances modify its treatment?

Dr. TAYLOR remarked, that no subject could come before the Society, for discussion, more important than the one now proposed. It was as important to the dental practitioner to properly understand the pathological conditions of the organs he treats, as for the practitioner of medicine to understand the pathological condition of the general system produced by functional derangement.

The address by our presiding officer, Dr. Hamill, fitly takes the initiative of this discussion; and as much involved in mystery as this subject may be, yet enough is known to form safe deductions therefrom. We know that dentine does take on inflammation—that this is governed very much by the condition of the general system—that general and local remedies will so counteract the inflamed state of dentine, that operations which would have been exceedingly painful, become of but little consequence to the patient.

In elucidation of his remarks, Dr. Taylor referred to an article published in the last No. of the Register, from the pen of S. James A. Salter, Esq., read before the Royal Medical Chirurgical Society.

The Doctor took up the ten propositions laid down by Mr. Salter, and commented on them separately in confirmation generally of the views there laid down. The reader is referred to January No., 1856, of Register, page 164.

Dr. GRIFFITH remarked, that there were two characters of ossification in diseased teeth. Where bone had been formed over the nerve he considered it unadvisable to disturb it. It was time enough to cut this formation away when the nerve was exposed. He scraped out all soft matter, but all that was hard enough to chip, he thought it better to leave undisturbed. He had filled hundreds of teeth under such circumstances, and in this way, and they always did very well.

Dr. RICHARDSON said, that in persons of advanced age there was a greater deposit of calcareous matter, and a greater tendency to ossification, which impaired, to some extent, the vitality of the teeth.

The increased sensibility at the point of junction of the enamel and dentine of teeth which had been remarked upon, found an analogy in the greater sensibility of the body immediately beneath the cuticle.

Dr. TAFT regretted that so little was known on this subject. There were, however, a few well-ascertained points from which we could safely reason in regard to it. There was an analogy between the teeth and the body. They had a circulation and ramification of nerves as the body had, and they partook of the peculiarities of various bodily diseases. Hereditary predispositions of constitution affected the teeth, and their vigor always corresponded with that of the general system. When there was inflammatory diathesis in the general system, the injured parts of the teeth would be affected, when at other times they would not.

These were general facts, but there were many minute and particular ones. Many times teeth decayed without any inflammation. Independent of the nerve, minute points of great sensibility would frequently be found in cavities where the walls were not at all sensitive. The nerves of a tooth did not pass into the enamel, but terminated between it and the dentine, and at the termination of nerves exalted sensibility was always found. Sometimes this sensibility was confined to a wall of laminæ about the cavity—then again the entire body of the dentine was found inflamed. Sometimes in cutting off the crown of a tooth it was found extremely sensitive, even before getting down to the nerve.

But under all these affections, the moment the nerve was destroyed all sensibility ceased, showing that it all arose from the nerve pulp in the center of the tooth.

EVENING SESSION.

Dr. HORTON, in filling teeth, had found, that bone of a hard, opaque appearance, or of a redish one, could always be cut out with impunity. A lighter shade indicated very little inflammation, but when very white, it was always very sensitive. He related a case of sensitiveness in a tooth after the nerve was destroyed. After destroying the nerve, he took it out in the evening, without pain to the patient, but it was too late to fill the tooth that day. The next day the patient came in and sat down to have it filled, not dreaming of any pain, but the moment the instrument was inserted to cleanse it, he started as if it had touched the nerve. On examination, a portion of the wall of the cavity looked very red, and appeared to be bleeding. But it was not—it was only the inflammation; for he could not get the least stain of blood upon his cotton. He would ask Dr. Taft how that sensitiveness, after the nerve had been removed, was to be accounted for?

Dr. TAFT—It had not been entirely removed—a part of it must have remained.

Dr. HORTON was sure the nerve had *all* been removed.

Dr. TAYLOR related an instance of extreme sensitiveness in a patient, where the nerve had been entirely destroyed.

The patient believed that the introduction of the instrument hurt him when it was not possible. He took pains to show him the extent of the cavity, and how far the instrument had been previously introduced, and after that he experienced no pain. It was all the effect of imagination—the action of the sympathetic nerve on the brain—for he felt assured that when the nerve was destroyed, all sensibility must cease. It could not be otherwise.

Dr. HORTON rose to state, with greater particularity, the circumstances of his case. He was certain the nerve had all been removed, and equally sure that his patient felt actual pain when he introduced his instrument. His patient was not, by any means, a timid, nervous, or imaginative man.

Dr. WATT thought the pain came from the nerve that originally supplied that tooth. Pain was really felt there, and imagination placed it not where the nerve then stopped, but where it had originally terminated. He related an instance, in which be had removed the nerve from a right central incisor. He cut the top of the tooth off, had a good opportunity to remove it all, and was quite sure he had done so. On the second call of the patient, the introduction of the instrument produced intense pain. She was a timid, nervous lady, and she accounted for it by saying, "Well, I was always very nervous, and I expect the nerve has grown out again."

When a nerve was cut off, inflammation took place at the point of separation, and caused pain, which was felt, not where it originated, but where the nerve had recently terminated. The patient's imagination had not yet accommodated itself to the new condition of the nerve.

Dr. ULREY would ask if it was not possible to cut off the nerve and leave live ramifications of it through the dentine? Teeth were well known to be highly vascular bodies, full of life. That blood circulated through them, we all knew, for it was sometimes forced in, in such quantities as to cause discoloration. Indeed, they were so full of vitality, that the great and almost the only effectual method of destroying it was by *poisoning* the life out.

Dr. GRIFFITH had invariably found the tooth sensitive immediately after the nerve had been destroyed. All inflammation could not be immediately destroyed by killing the nerve. At the apex of the fang sensibility would remain, because the great arterial trunk was there. As acids acted readily on the dentine, a soreness would remain as the consequence of their use. A good remedy for this he had found in the bi-carbonate of soda, and had advised his patients to use it in such cases, to dstroy the *effect* of the acids.

Dr. JOS. TAYLOR would ask: If ramifications of the nerve did remain in the dentine, how could they communicate pain when totally disconnected at the point of the tooth? You could not communicate pain from a dead body to a living body without a connection.

Dr. Griffith would answer this by asking another question: Was not the tooth supplied from other quarters? The life of a tooth might not be entirely destroyed even when the nerve was destroyed.

Dr. Jos. Taylor had never, in thirty years practice, found any tenderness in the actual dentine of the crown after the nerve had been destroyed. The most he had ever found was sometimes a little sensibility at the root.

Dr. James Taylor said: It had been demonstrated that the pulp was not always dead when the nerve was cut off at the point. He related an instance, in which he had extracted a tooth where there had been a separation at the point, and on splitting it open, he found it contained blood. The circulation seemed to have been kept up through the periosteum. It was important to know whether this was a vital circulation. He hoped the members of the profession would take the pains to split open and examine such teeth whenever they extracted them.

Dr. Taft thought, as attention had been directed to this subject, the members would probably all know more of it next year. He hoped, therefore, that the question would not be disposed of at this time. This hope was also expressed by other members, and the discussion of the question suspended here with an understanding that it should be recommended for discussion next year.

Subject No. 10 was then taken up and discussed.

Question, 10. What is the modus operandi of Arsenic, Chloride of Zinc, Nitrate of Silver, Creosote, Tannin, etc., when applied to inflamed Dentine?

Dr. Bonsall thought that tannin, being an astringent, and having a different character from all the other agents named, ought not to be classed with them.

Dr. Ulrey thought it enough to say of most of them, that they were poisons, and the manner of their action was simply to poison the life out of the bone. There was always danger that arsenic would destroy the tooth. If left in a little too long, it was sure to do so.

Dr. WATT said, that nearly all these agents, or the majority of them, at least, were of the class known as escharotic remedies. Their tendency was to produce the death of the part—which tendency was counteracted by the vital action. We all knew that nothing but the vital force of the stomach protected it from the gastric fluid. The loss of vitality in one part, however, always produced a change of action in the surrounding parts.

Escharotics were divided into two classes—those which were absorbed and passed into the system, and others which were not. Arsenical and mercurial preparations were liable to absorption, while chloride of zinc was not.. He never applied arsenic to inflamed dentine, for, after using it, such was the appearance of the tooth that he never could have any means of knowing whether he had excavated all the affected parts or not.

[The first part of Dr. Watt's remarks embraced a careful chemical analysis and description of all the agents mentioned, but abounded so much in scientific and professional terms that the reporter was not able to follow him with any accuracy.]

Dr. RICHARDSON spoke of the difficulty of knowing the extent of the effect of the arsenical preparation, and related an instance in which he had probably not excavated sufficiently.

Dr. WATT thought no definite line of absorption could be found.

Dr. TAFT thought it was impossible to tell anything about the absorption of arsenic. It could reach the periosteum in two ways—either by the walls of the canal or by being taken up by the pulp. The rapidity with which it was taken up would depend upon the action of the fluids. In young persons, where the tooth was very vascular, it was taken up with great rapidity, and the tooth frequently destroyed. He seldom used arsenic for this or any other purpose except to poison rats.

Chloride of zinc or nitrate of silver left the adjacent parts healthier than before, but arsenic destroyed vitality as far as it went.

Dr. TAYLOR could not agree with the gentlemen in all of the positions taken. He, however, never used arsenic in inflamed dentine. He did not think inflamed dentine ever ought to be treated with any such preparation.

He had wished very much that some substitute could be found for creosote, which he was using more and more every day. He valued it very much for its antiseptic properties.

Dr. GRIFFITH had used arsenic extensively, and from the experience he had had with it he intended to continue to do so. He claimed to have no chemical knowledge of it, and had therefore no prejudice against it. All his knowledge of it was derived from experience. It was true, it was a poison, but there were antidotes for all poisons. As beneficial as he had found it in his practice, he well knew that it might easily be used in such a way as to destroy the tooth. He had used it for the last eighteen years, and in more than a thousand teeth—had always employed it with charcoal and creosote, and always with success.

A number of the members having expressed a desire to hear subjects 9 and 10 more fully discussed, they were, on motion, referred to the Executive Committee, with a recommendation to place them on the order of business for the next annual meeting.

By request, Dr. GODDARD then gave an account of an interesting and singular case of excrescence of the gum, for which he operated, in the Hospital, at Louisville; but as the Doctor, to gratify the wishes of a number of the members, engaged to write a full account of it for the *Dental Register*, it is not necessary to give the facts in regard to this very curious case here.

